# A rabbit model of right-sided *Staphylococcus aureus* endocarditis created with echocardiographic guidance

**DOI:** 10.1186/1476-7120-11-3

**Published:** 2013-01-14

**Authors:** Mei-lian Wang, Ying Zhang, Miao Fan, Ya-jun Guo, Wei-dong Ren, En-jie Luo

**Affiliations:** 1 Department of Microbiology and Parasitology, College of Basic Medical Sciences, China Medical University, No. 92 Beier Road, 110001, Heping District, Shenyang, China; 2 Department of Sonography, Shengjing Hospital of China Medical University, No. 36 Sanhao Street, 110004, Heping District, Shenyang, China; 3 Department of Radiology, The First Affiliated Hospital of Sun Yat-sen University, 510080, Guangzhou, China

**Keywords:** Infective endocarditis, Echocardiography, Catheterization, Animal model

## Abstract

**Background:**

The most widely used experimental models of infective endocarditis (IE) are the rabbit and rat models, in which cardiac valve lesions are induced by a polyethylene catheter introduced into the left ventricle through the aortic valve. Our study was designed to create a rabbit model of IE right-sided with echocardiographic guidance.

**Methods:**

Thirty rabbits underwent both catheterization and inoculation (group A). These were divided into three subgroups of ten based on the time of catheter removal (immediately, after 24 h, and after death or moribundity for groups, A_1_, A_2_, and A_3_, respectively). Ten inoculated-only and ten catheterized-only rabbits served as controls. A catheter system consisted of a polyethylene catheter and a guide wire inside it. This system was passed through the rabbits’ tricuspid valves under echocardiographic guidance to damage them. The ratio of left ventricle to right ventricle (LV/RV) was measured in a four-chamber view before catheterization and at the time of death or moribundity. The peak velocity of tricuspid regurgitation (V_TR_) was measured in a four-chamber view at the time of catheterization and at the time of death or moribundity. *Staphylococcus aureus* (ATCC 29213) inoculation was performed 24 h after right heart catheterization to produce IE.

**Results:**

IE was confirmed in 28 of 30 rabbits by macroscopic and histologic examination of tricuspid valves, blood cultures, and bacterial count in cardiac vegetations. Cardiac vegetations were confirmed in 25 of 28 IE rabbits by echocardiography. Enlargement of right ventricle dimension with a significantly decreased LV/RV ratio was confirmed in all IE rabbits at the time of death or moribundity than at the initial state (1.11 ± 0.35 vs. 1.95 ± 0.39, *P* < 0.01; 1.21 ± 0.34 vs. 1.98 ± 0.35, *P* < 0.01; 1.04 ± 0.31 vs. 2.00 ± 0.41, *P* < 0.01 for groups A_1_, A_2_, and A_3_, respectively). V_TR_ was significantly higher in all the IE rabbits at the time of death or moribundity than at the time of catheterization (1.89 ± 0.46 vs 0.76 ± 0.45, *P* < 0.01; 2.04 ± 0.73 vs 0.68 ± 0.66, *P* < 0.01; 2.24 ± 0.51 vs 0.87 ± 0.55, *P* < 0.01 for group A_1_, A_2_ and A_3_, respectively).

**Conclusion:**

The models described herein closely reproduced the pathogenesis and pathophysiology of right heart catheter-induced endocarditis in humans. Echocardiographic guidance is helpful in the process of right heart catheterization. Some echocardiographic parameters, such as V_TR_ and the LV/RV ratio could be used to assess the success or failure of the IE models.

## Background

Infective endocarditis (IE) is a rare disease that may lead to serious consequences for patients. According to the location of infection, IE is usually classified into four categories: native valve endocarditis (NVE), prosthetic valve endocarditis (PVE), right-sided endocarditis and device-related endocarditis [1]. Viridans group streptococci and staphylococci are most often isolated from blood cultures in IE.

Three fundamental methods are applied in experimental models of IE. The valves of larger animals, such as dogs, are damaged by extensive surgical procedures to allow subsequent bacterial colonization [2,3]. This model is not fully reliable due to the high mortality rate caused by surgical injury. A catheter-related model for IE has seen widespread use since it was first described in a report [4] by Garrison and Freedman. Usually, the catheter is introduced into the left ventricle [5,6] through aortic valve or introduced into the right ventricle [4,7] through tricuspid valve, followed by intravenous injection of bacteria, to produce a left-sided or right-sided IE model. The processes of catheter introduction were either unguided, although some were confirmed by angiography. These models are unreliable because the valve may remain undamaged if the catheter is placed in the wrong place. The presence of an intracardiac foreign body, in this case an indwelling catheter, represents an obvious deviation from the human situation. These models more closely mimic pathogenesis of PVE [8]. Recently, Maurin established a guinea pig model that used electrocoagulation of native aortic valves to induce lesions in the endothelium without the need for an indwelling intracardiac catheter [9,10]. This model has proven itself valuable, especially in its close resemblance to the pathogenesis of NVE, but the complicated procedure limits its use.

With the introduction and development of modern invasive diagnostic and therapeutic techniques, it has been recognized that cardiac catheterization is closely related to the incidence of IE, mainly on the right side of the heart [11,12]. Our study was designed to develop an experimental model of right-sided IE, which had similar pathogenesis to right heart catheter-induced endocarditis in humans. A catheter assembled with a guide wire was designed to get through the tricuspid valve precisely and produce the lesions on the valve under echocardiographic guidance.

## Methods

### Ethics statement

All animal procedures were approved by the Animal Ethics Committee of China Medical University and were conducted in compliance with institutional regulations.

### Animal treatment

Fifty New Zealand white rabbits (25 males and 25 females), weighing 2–2.5 kg, were used in the study. Animals were randomly assigned to three groups. In group A, which contained 30 rabbits, *Staphylococcus aureus* (ATCC 29213) inoculation was performed 24 h after right heart catheterization. This group was divided into three subgroups of ten rabbits each according to the time at which the catheter was removed. The rabbits from which the catheter was removed immediately or 24 h after right heart catheterization are called groups A_1_ and A_2_, respectively. The other ten rabbits, in which the catheters were kept in the right heart until death, are called group A_3_. In group B, ten inoculated-only controls were inoculated but not subjected to right heart catheterization. In group C, right heart catheterizations were performed in uninoculated 10 controls. All the rabbits were kept in individual cages and given water and commercial food *ad libitum*.

### Right heart catheterization

The animals were anesthetized with pentobarbital sodium (30 mg/Kg ip). Their chests and bilateral inguinal regions were shaved. An incision was made in the left inguinal region where pulsation could be felt by touch. The left femoral vein (LFV) was then exposed and dissected. A polyethylene catheter with an external diameter 0.8 mm and internal diameter 0.4 mm was used to perform the right heart catheterization. A steel guide wire 0.2 mm in diameter with bent tips was introduced into the cavity of the catheter. When it penetrated into the LFV and was passing down the vein into the right atrium, the tip of the guide wire aligned with the catheter. The catheter system containing the guide wire was then introduced into the LFV and the right atrium through a median anterior incision. When the end of catheter was visualized at the entrance of the right atrium under echocardiographic guidance, the guide wire was pushed forward until about 1 cm of the tip was exposed. Adjust the direction of the catheter and the guide wire to allow the catheter system to pass through the tricuspid valve. In the process of catheter introduction, the external wall and the lumen of the catheter were flushed with heparinized sterile saline solution continuously. The time required to complete the process was recorded.

### Echocardiographic guidance and measurement

An ultrasound system (Philips iE 33), equipped with a 4–12 MHz transducer was used in this study.

Before catheterization, the sizes of the left and right ventricles were measured. The diameter of the left and right ventricles were measured at the level of atrioventicular valve annulus and at the middle of the two ventricles, respectively. The average dimensions of the two ventricles were used to calculate the ratio of left ventricle to right ventricle (LV/RV). The measurements were repeated at the time the animal was killed or moribund. The LV/RV ratio in different periods was recorded for later analysis.

**Figure 1:**
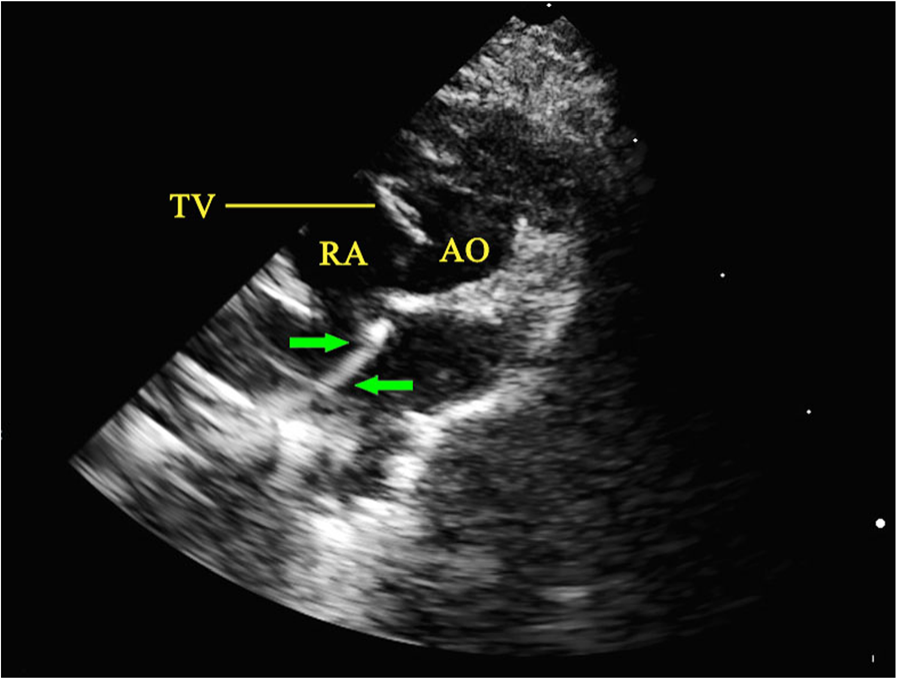
**Entry of the catheter system into the right atrium as visualized by echocardiography. **The catheter system was visualized entering the right atrium, close to the atrial septum in aortic short axis view by echocardiography. The green arrows indicate the position of the catheter system. AO, aorta; RA, right atrium; TV, tricuspid valve.

**Figure 2:**
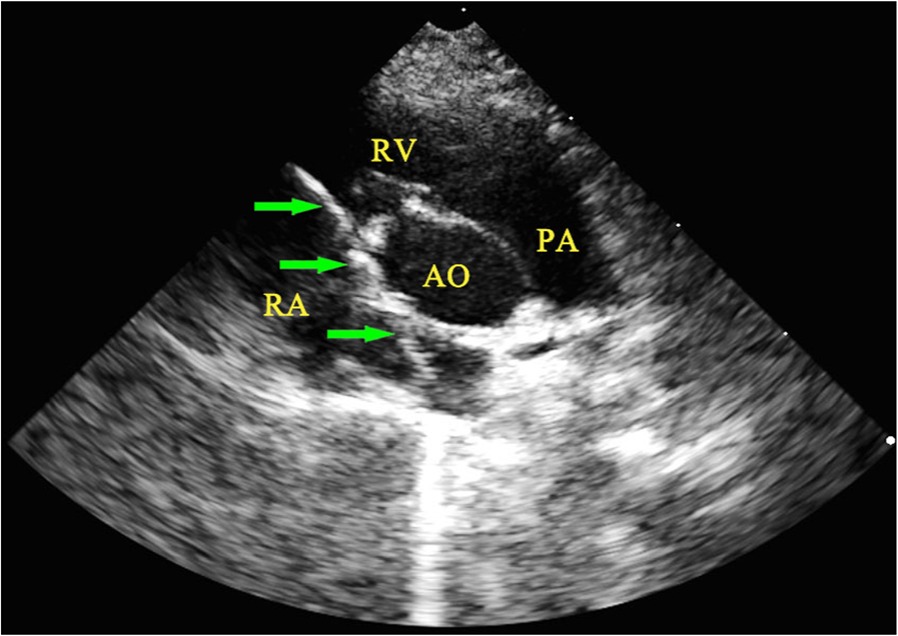
**Passage of the catheter system through the tricuspid valve as visualized by echocardiography. **The passage of the catheter system through the tricuspid valve and entering the right ventricle was visualized in aortic short axis view by echocardiography. The green arrows indicate the position of the catheter system. AO, aorta; PA, pulmonary artery; RA, right atrium; RV, right ventricle.

**Figure 3:**
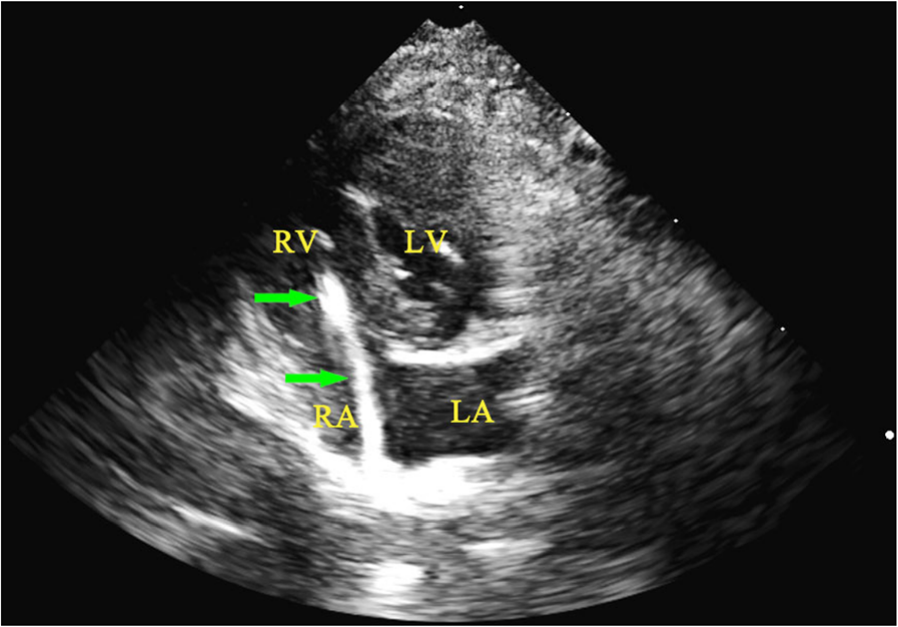
**Confirmation the position of the catheter system by echocardiography. **The position of catheter system, passing through the tricuspid valve, was confirmed by echocardiography in a four-chamber view. LA, left atrium; LV, left ventricle; RA, right atrium; RV, right ventricle.

During catheterization, an aortic short axis view was acquired when the end of the catheter was visualized at the entrance of the right atrium (Figure 1). Under echocardiographic guidance, the catheter system was passed through the tricuspid valve (Figure 2). The catheter system was moved forward and backward repeatedly to damage the tricuspid valve. As the tip of guide wire bent, the tricuspid valve could be damaged easily. A four-chamber view was then created to confirm the position of the catheter system (Figure 3) and to assess the degree of tricuspid regurgitation. Usually, the regurgitation could be identified after the catheter systems had passed forward and backward through the tricuspid valve three to five times. The peak velocity of tricuspid valve regurgitation (V_TR_) was recorded. The measurements were repeated at the time the animal was killed or moribund.

Echocardiography was also performed in the rabbits when they were killed or moribund to confirm the position and size of cardiac vegetations by aortic short axis and four-chamber views.

All the echocardiographic views were saved as video clips.

### Production and confirmation of IE

The rabbits were injected with *S. aureus* (ATCC 29213) suspension (0.5 ml, i.e., 8×10^7^ CFU) through ear marginal vein. The body temperature was recorded during the challenge. Blood cultures were performed by intracardiac puncture at the time of rabbit death. The presence of IE was confirmed by macroscopic and histologic examination of the cardiac valves. Tricuspid valves were excised and prepared for light microscopy. After fixation in 10% formol saline, the specimens were embedded in paraffin and sectioned at 5 μm. These specimens were then stained with hematoxylin-eosin.

### Assessment of tricuspid valve damage

Valve lesions of rabbits in group C were assessed by macroscopic and histologic examination of the heart valves as described above.

### Quantitative microbiologic analysis

Bacterial titers per gram of tissue were determined in cardiac vegetations from the rabbits with IE. The tissue fragments were crushed with tryptic soy broth (Sigma, U.S.), and 10-fold serial dilutions of each were inoculated onto tryptic soy agar plates. Bacterial titers were determined in terms of CFU.

### Statistical analysis

Data are expressed as means ± SD. A paired Student *t*-test was used to compare continuous variables, with *P <* 0.05 considered indicative of statistical significance. All statistical analyses were performed using commercially available software (SPSS, release 17.0).

## Results

The mean time for right heart catheterization (from the time of incision of the LFV to the time the catheter completed its passage through the tricuspid valve) was 15 ± 2 min.

Of the 30 rabbits in group A, ten died spontaneously. None of the rabbits in group B and only 1 rabbit in group C died spontaneously. The body temperature in all inoculated rabbits increased to at least 40°C during the 72 h following inoculation. No discernible difference was observed between rabbits in group A and group B and no febrile response was confirmed in rabbits of group C.

Histologic examination of tricuspid valves of the 10 rabbits in group C after right heart catheterization without inoculation demonstrated endothelial lesions and showed localized inflammation of valvular tissue sometimes associated with small fibrin deposits. It presented nonbacterial thrombotic vegetations (NBTV) on the endothelial surface.

**Figure 4:**
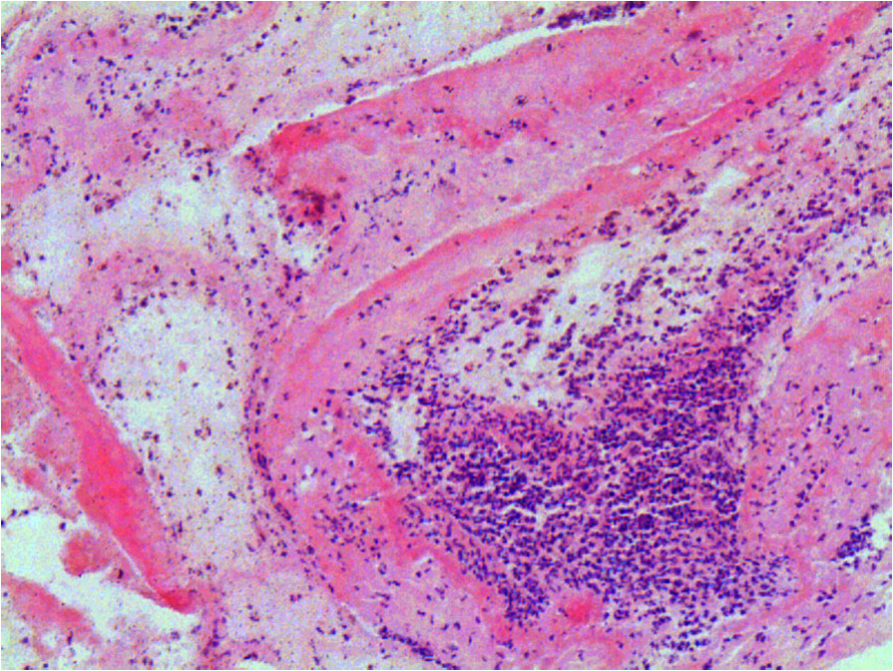
**Histologic examinations of the infectious vegetations. **Histologic examinations showed large infectious vegetations with extensive destruction of valve tissue. Heavy inflammation was also found in the tissue with appearance of large numbers of neutrophils (HE × 100).

Macroscopic and histologic examination of the tricuspid valves demonstrated the presence of IE in 28 rabbits (8, 10, and 10 rabbits for groups A_1_, A_2_, and A_3_, respectively), all of which had both undergone catheterization and inoculation. Vegetations about 5–10 mm, adhering to valves, were visualized under macroscopic examinations. Usually, the vegetations were yellow or gray-white. Histologic examinations showed large infectious vegetations with extensive destruction of valve tissue. Heavy inflammation was also found in the tissue with appearance of large numbers of neutrophils (Figure 4).

Blood cultures were positive for 27 of the 30 rabbits in group A. All died spontaneously or were killed on day 5. The mean bacterial count of the cardiac vegetations was measured for the rabbits in group A. All died spontaneously or were killed on day 5. The results are summarized in Table 1.

**Table 1:** Comparison of spontaneous deaths, pathologic findings, blood cultures, and mean bacterial count in cardiac vegetations among the rabbits of the three groups in our study

**Experimental design**	**Group A (n = 30)**			**Group B (n = 10)**	**Group C (n = 10)**
	**A**_**1 **_**(n = 10)**	**A**_**2 **_**(n = 10)**	**A**_**3**_** (n = 10)**		
Spontaneous death	2	3	5	0	1
Endocarditis	8	10	10	0	0
Positive blood culture	7	10	10	NA	NA
MBC in cardiac vegetations	3.5×10^9^ ± 3.9×10^9^	4.9×10^9^ ± 5.5×10^9^	5.8×10^9^ ± 6.9×10^9^	NA	NA
NBTV	NA	NA	NA	0	10

group A: rabbits underwent both catheterization and inoculation; group A_1_: the catheter was removed immediately after catheterization; group A_2_: the catheter was removed 24 h after catheterization; group A_3_: the catheter was kept in the right heart until rabbit death; group B: rabbits only inoculated; group C: rabbits only underwent catheterization; *MBC*: mean bacterial count; *NBTV*: nonebacterial thrombotic vegetation; *NA*: not applicable.

**Figure 5:**
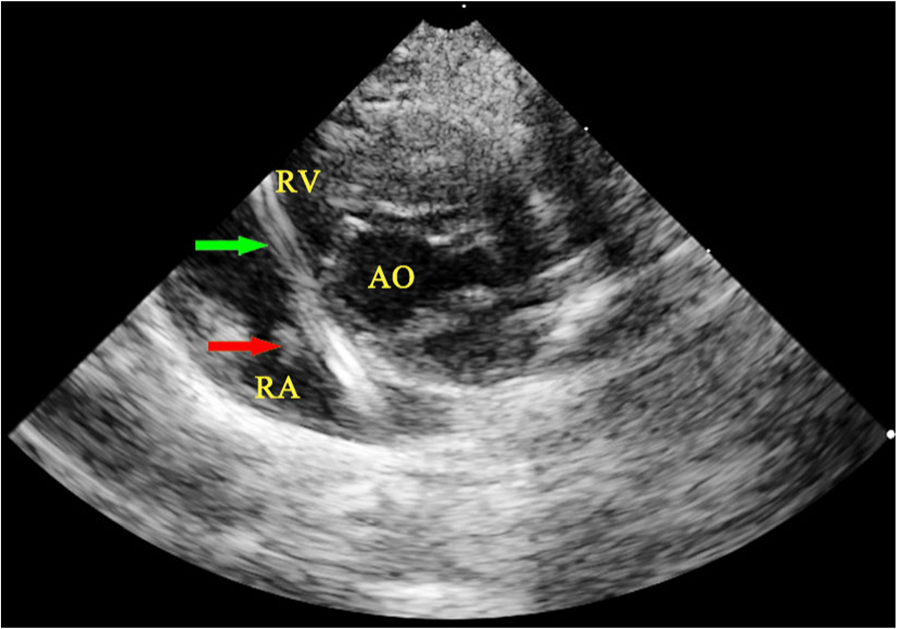
**Vegetations visualized by echocardiography. **Vegetations (indicated by the red arrow) were clearly visualized by echocardiography in a rabbit in Group A3. The green arrows indicate the position of the catheter system. AO, aorta; RV, right ventricle.

Echocardiography examinations showed that the LV/RV ratio was about 2:1 in all the rabbits before any treatments were made. No tricuspid regurgitation was observed in any of the rabbits. Enlargement of the right ventricle was observed in all IE rabbits in groups A_1_, A_2_, and A_3_ and the ratio of the two ventricles remained unchanged in all the non-IE rabbits. At the time of catheterization, tricuspid regurgitation was visualized in all the rabbits in groups A and C. V_TR_ was significantly higher in all the IE rabbits at the time of death or moribundity than at the time of catheterization. In contrast, V_TR_ remained unchanged in the non-IE rabbits in groups A and C. Vegetations were clearly identified by echocardiography (Figure 5) in 25 of 28 IE rabbits in group A (Table 2).

**Table 2:** Echocardiographic findings in rabbits

**Experimental design**	**Group A**_**1**_		**Group A**_**2**_	**Group A**_**3**_	**Group B**	**Group C**
	**IE (8)**	**Non-IE (2)**	**IE (10)**	**IE (10)**	**None-IE (10)**	**None-IE (10)**
LV/RV ratio (initial)^state a^	1.95 ± 0.39	2.10 ± 0.42	1.98 ± 0.35	2.00 ± 0.41	2.04 ± 0.36	2.23 ± 0.35
LV/RV ratio (later) ^state b^	1.11 ± 0.35^*^	1.95 ± 0.50	1.21 ± 0.34^*^	1.04 ± 0.31^*^	2.11 ± 0.24	2.29 ± 0.38
V_TR_ (m/s) (initial) ^state c^	0.76 ± 0.45	0.45 ± 0.21	0.68 ± 0.66	0.87 ± 0.55	NA	0.52 ± 0.39
V_TR_ (m/s) (later) ^state b^	1.89 ± 0.46^#^	0.40 ± 0.14	2.04 ± 0.73^#^	2.24 ± 0.51^#^	NA	0.47 ± 0.15
Detection of vegetations	7	0	9	9	0	0

^state a^: measured before any treatment; ^state b^: measured at the time of death or moribundity; ^state c^: measured at the time of catheterization; ^*^: *P* < 0.01 state b vs. state a; ^#^: *P* < 0.01 state b vs. state c; *IE*: infective endocarditis; *LV*: left ventricle; *RV*: right ventricle; *V*_*TR*_: peak velocity of tricuspid regurgitation.

## Discussion

IE has been a great challenge to routine clinical practice for decades. This is due to many factors, such as poor penetration of antibiotics into vegetations, altered metabolic state of bacteria within the lesion, and the absence of adequate host-defense cellular response capable of cooperating with antibiotic action [13]. Animal models of IE enables a better understanding of the pathogenesis and pathophysiology of the infection by helping to improve the therapeutic and prophylactic regimens of IE in humans.

There are several animal models of IE, most in rabbits or in rats. The most widely used are the polyethylene catheters introduced in the left ventricle [5,6]. The catheter remains in the heart for 24 h or until the animal’s death. Under these circumstances, the catheter acts mainly as a foreign body in the heart. The pathogenesis of IE in these models corresponds to that of PVE [8]. However, the intracardiac catheter may affect the ability of bacteria to colonize the endocardium [14-17]. This may impede the pathogenesis of PVE in some extent. The intracardiac catheter may decrease the efficacy of the antibiotic therapy used for either therapeutic or prophylactic purposes [18]. This restricts the use of this model in many respects.

With the development of modern invasive diagnostic and therapeutic techniques, IE complicated by right heart catheterization, such as right heart catheter angiography, atrial septal defect occlusion, placement of intracardiac pacemaker, and other conditions, have become more common in clinical practice. We described an experiment meant to establish whether it was possible to produce right-sided IE in rabbits using a polyethylene catheter combined with an internal guide wire. This condition had similar pathogenesis to right heart catheter-induced endocarditis in humans. Rabbits in group A_1_ were made to resemble models of right heart catheter-induced IE, and rabbits in groups A_2_ and A_3_ resembled models of intracardiac foreigners producing PVE, similar to previous reports [4-7]. The results in our study showed a good consistency in production of IE in the three subgroups.

In our reports, most of the IE rabbits showed positive blood cultures, demonstrating active endocarditis. IE was confirmed by macroscopic and histologic examinations of tricuspid valves and vegetations. The numbers of bacteria within cardiac vegetations were comparable to those obtained in rabbits and rats in previous reports [4-6,19]. Both the NBTV and the S. *aureus* vegetation in the hearts of the rabbits bore a striking resemblance to human IE and embolic phenomena in rabbits with IE were consistent with pathologic data on humans [20,21].

Unlike in previous studies, a catheter system with a guide wire inside was used to make valve lesions. The catheter acted mainly as a carrier to transport the guide wire into its position at the tricuspid valve. The guide wire had two main roles. First, it guides the catheter systems into position. The inferior vena cava drains into the right atrium pointing toward the atrial septum. The catheter system must rotate 90° to point to the tricuspid valve when it enters the right atrium. As the tip of the guide wire bends, it easily causes the catheter system to rotate into the tricuspid valve when the guide wire encounters the atrial septum. In previous studies, polyethylene catheters were introduced into the right side of the heart via the femoral vein in a rabbit model [4,7]. In these reports, only a few catheters were found to pass through the tricuspid valve, but, in many cases, the catheters were located at the right atrium, or even beneath the pericardium. This showed the difficulty of moving the catheter toward the tricuspid valve without the help of a guide wire. Second, tricuspid valve lesions are made by the tip of the guide wire. In all the rabbits in group C in the present report, NBTV were observed during histologic examinations. Valve lesions made by the guide wire ensured the production of IE when the catheter systems were removed immediately after catheterization (group A_1_).

Echocardiographic guidance of catheterization was another highlight of our study. This differed from techniques used in previous studies in that previous catheter introduction processes were either unguided, though some of them were confirmed by angiography. Echocardiographic guidance ensured that the catheter system would pass through the valve precisely, making the desired valve lesions, and move over, in turn ensuring the reliability of the models. Despite this, echocardiographic guidance did not increase time requirements for the procedures. This was because one operator manipulated the introduction of the catheter system while the other made the echocardiographic examination. The mean time of the procedures in our reports was 15 min, even shorter than the unguided procedures described in previous reports [4] 20 min. If the catheterization was guided by angiography, the estimated time for X-ray exposition was about 1 to 1.5 min for one rabbit. Because echocardiographic examination costs much less than angiography and does not expose the subject or clinicians to additional radiation damage, echocardiographic guidance may be preferable to other methods in the production of IE models.

Some echocardiographic parameters were evaluated in our study. Tricuspid regurgitation appeared in all the confirmed IE rabbits, followed by enlargement of right ventricle. We speculated that tricuspid valve lesions made by the guide wire caused the tricuspid regurgitation, which was aggravated by infection with *S. aureus*. Tricuspid regurgitation also led to gradual enlargement of the right ventricle. The extent of tricuspid regurgitation and enlargement of the right ventricle were positively related to the occurrence of IE in the rabbit model of the present study. We believe that some echocardiographic parameters, such as V_TR_ and the LV/RV ratio, could be used to assess the success or failure of the IE models we produced. It also confirmed the value of our study.

### Study limitations

The present study has some limitations. We did not set up a group in which the catheter introduction process was performed without guidance. In this way, we could not accurately evaluate the difference (including time required for catheterization and success rate of IE) between our method (echocardiographic guidance) and the traditional method in producing right-sided IE. We only compared the corresponding data in our study to those in previous report [4]. Our goal was to use as few animals as possible to establish a reliable animal model relevant to right-sided IE.

## Conclusion

In conclusion, the models described herein closely reproduce the pathogenesis and pathophysiology of right-heart catheter-induced endocarditis and may allow more relevant investigations of pathogenesis and pathophysiologic mechanisms of right-sided IE in humans. Echocardiographic guidance proved its advantages over previous methods in the process of right heart catheterization. Some echocardiographic parameters, such as V_TR_ and the LV/RV ratio could be used to assess the success or failure of the IE models.

## Abbreviations

IE: Infective endocarditis;LV: Left ventricle;NBTV: Nonbacterial thrombotic vegetations;NVE: Native valve endocarditis;PVE: Prosthetic valve endocarditis;RV: Right ventricle;VTR: Peak velocity of tricuspid regurgitation

## Competing interests

The authors declare that they have no competing interests.

## Authors’ contributions

M-lW and YZ designed the whole study. M-lW, YZ, MF, Y-jG, W-dR, and E-jL drafted the manuscript. YZ and Y-jG performed right heart catheterization and echocardiographic examinations. M-lW performed the microbiologic experiments. All authors have read and approved the final manuscript.
